# The diversity of hydrogen-producing bacteria and methanogens within an in situ coal seam

**DOI:** 10.1186/s13068-018-1237-2

**Published:** 2018-09-08

**Authors:** Xianbo Su, Weizhong Zhao, Daping Xia

**Affiliations:** 1 School of Energy Science and Engineering, Henan Polytechnic University, Jiaozuo, 454000 China; 2 Collaborative Innovation Center of Coalbed Methane and Shale Gas for Central Plains Economic Region, Jiaozuo, 454000 Henan Province China

**Keywords:** Coalbed methane, Biogenic, Environment factors, Diversity, Hydrogen-producing bacteria, Methanogens

## Abstract

**Background:**

Biogenic and biogenic-thermogenic coalbed methane (CBM) are important energy reserves for unconventional natural gas. Thus, to investigate biogenic gas formation mechanisms, a series of fresh coal samples from several representative areas of China were analyzed to detect hydrogen-producing bacteria and methanogens in an in situ coal seam. Complete microbial DNA sequences were extracted from enrichment cultures grown on coal using the Miseq high-throughput sequencing technique to study the diversity of microbial communities. The species present and differences between the dominant hydrogen-producing bacteria and methanogens in the coal seam are then considered based on environmental factors.

**Results:**

Sequences in the Archaea domain were classified into four phyla and included members from *Euryarchaeota*, *Thaumarchaeota*, *Woesearchaeota*, and *Pacearchaeota*. The Bacteria domain included members of the phyla: *Firmicutes*, *Proteobacteria*, *Bacteroidetes*, *Actinobacteria*, *Acidobacteria*, *Verrucomicrobia*, *Planctomycetes*, *Chloroflexi*, and *Nitrospirae.* The hydrogen-producing bacteria was dominated by the genera: *Clostridium*, *Enterobacter*, *Klebsiella*, *Citrobacter*, and *Bacillus*; the methanogens included the genera: *Methanorix*, *Methanosarcina*, *Methanoculleus*, *Methanobrevibacter*, *Methanobacterium*, *Methanofollis*, and *Methanomassiliicoccus.*

**Conclusion:**

Traces of hydrogen-producing bacteria and methanogens were detected in both biogenic and non-biogenic CBM areas. The diversity and abundance of bacteria in the biogenic CBM areas are relatively higher than in the areas without biogenic CBM. The community structure and distribution characteristics depend on coal rank, trace metal elements, temperature, depth and groundwater dynamic conditions. Biogenic gas was mainly composed of hydrogen and methane, the difference and diversity were caused by microbe-specific fermentation of substrates; as well as by the environmental conditions. This discovery is a significant contribution to extreme microbiology, and thus lays the foundation for research on biogenic CBM.

**Electronic supplementary material:**

The online version of this article (10.1186/s13068-018-1237-2) contains supplementary material, which is available to authorized users.

## Background

Coalbed methane (CBM) is an important energy reserve of unconventional natural gas. As new energy sources continue to develop, the exploitation and utilization of CBM is inevitable. Moreover, the use of CBM might improve the structure of energy supply, alleviate the crisis caused by energy shortages, improve effective mining conditions, and meet the demand for low carbon environmental protection. Most early studies on the origin of CBM agree that CBM is primarily of thermogenic and biogenic origin. Some of the world’s CBM reservoir is biogenic, such as the Powder River Basin in the U.S. and the Surat Basin in Australia [1, 2]; and some are both thermogenic and biogenic, such as the San Juan basin [3], the Sydney basin [4], Hokkaido, Japan [5], and some blocks of the Qinshui basin in China [6].

The stable isotope values (δ^13^C and δ^2^H for methane) and geochemical characteristics in various geological settings suggest an identification scheme of biogenic methane [7, 8]. In general, a value of − 55‰ provides guidance to distinguish the origin of the CBM; δ^13^C values less than − 55‰ indicate biogenic origin and values greater than − 55‰ indicate thermogenic origin. Furthermore, the δ^2^H value of CH_4_ is an isotopic indicator that distinguishes biogenic and thermogenic origins, where δ^2^H values less than − 250‰ indicate thermogenic gas and values greater than − 250‰ suggest biogenic gas. Several studies have indicated formation mechanisms of biogenic methane. They include acetic fermentation (CH_3_COOH → CH_4_ + CO_2_), carbon dioxide reduction (CO_2_ + 4H_2_ → CH_4_ + 2H_2_O) and methylotrophic reactions (4CH_3_OH → 3CH_4_ + HCO3^−^+H_2_O + H^+^) [9–11].

Anaerobic fermentation of organic matter into methane involves hydrolysis, acidogenesis, acetogenesis, and methanogenesis, all of which rely on the syntrophic interactions of microorganisms in all stages of fermentation [12]. The hydrolyzing bacteria in the primary stage of fermentation secrete extracellular enzymes, which catalyze the hydrolytic degradation of macromolecules into simple sugars, amino acids, and fatty acids. Bacterial acidogenesis further degrades simple organic matter into propionic acid, butyric acid, pentanoic acid, other volatile fatty acids (VFAs), and alcohols. Bacterial acetogenesis in the second stage of fermentation mainly converts VFAs into acetic acid, H_2_ and CO_2_. The final stage of methane formation is performed by methanogens, which are classified as acetoclastic, hydrogenotroph or methylotrophic, depending on which substrates were preferred by the previous fermentation [13].

As a kind of complex organic matter, the maximum conversion of coal to methane also requires the synergistic action of at least three groups of microorganisms as discussed above. First, from a thermodynamic perspective, the standard Gibbs free energy, ∆G^0′^, of ethanol, propionic acid, butyric acid, and valeric acid (other than lactic acid) is positive, and the reaction will not proceed spontaneously under standard conditions [14]. However, the literature has shown that ∆G^0′^ can become negative when the partial pressure of hydrogen is lower than 10^−5^ bar and the reaction will take place spontaneously [15–17]. Second, the mutual metabolism of hydrogen-producing bacteria and methanogens can eliminate the accumulation of intermediate hydrolysis products, and this provides power for the conversion of organic matter into methane [18]. In recent years, there has been an increase in the emphasis on coal-based biogas research. This increase has included laboratory studies of factors and mechanisms for the production of biomethane from coal [19–21], especially in regard to the bacteria from the mine water or CBM wells drainage water [21, 22]. Few studies use the hydrogen-producing bacteria and methanogens from the coal seam as the microbial source to carry out these biogas experiments. Therefore, the determination of bacterial and archaeal community structures of the coal seam would be of great importance for understanding microbial syntrophic interactions and conversion mechanisms of biomethane.

The coal seam is an extreme environment, i.e., coal seam is extremely anaerobic relative to the ground environment. Coal seam microorganisms may differ from the conventional species in their genetic makeup and physiological function. Therefore, some unknown and extensive biological cycles remain to be discovered. High throughput sequencing techniques are the most widely used new generation sequencing technology [23, 24]. These techniques have unique advantages in analyzing the microbial community structure. It is possible to build and sequence the gene library from the total DNA obtained directly from environmental samples. The key is to generate 16S rRNA gene data, which can be used to estimate the species composition of the microbial communities under study; as well as the complexity and diversity of microbial communities in environment [25, 26].

In 2007, Shimizu [5] reported, for the first time, the microbial diversity in a coal seam found in Hokkaido, Japan. Geochemical methods were used to measure the gas composition and stable isotopes in these samples. The bacterial and archaeal diversity of CBM water was determined from the 16S rRNA gene library and showed that *Methanoculleus* and *Methanolobus* are the dominant methanogens in that area. These results also confirmed the thermogenic and biogenic origin of this CBM. A systematic geochemical study by Strapoc et al. of the CBM field located east of the Illinois basin in the U.S. combined with analysis of the respective 16S rRNA gene library showed that the archaea and bacteria in this area were primarily *Methanocorpusculum* and *alpha*-*Proteobacteria*, *Firmicutes* and *Clostridium*, respectively [27]. Furthermore, the hydrogenotrophic (CO_2_ reduction) methanogens detected in this area suggest that the CBM is biogenic. Midgley et al. used a 16S rRNA gene library to detect the existence of bacteria and archaea in the drainage water of the CBM well in the Gippsland basin, Australia [28]. *Proteobacteria*, *Firmicutes*, and *Methanobacter* were major components of the microbial community from these drainage water samples. There are few studies reported of the microbial diversity of coal seams in China; however, coal seam microorganisms in different regions of the Ordos basin have been detected by the 16S rRNA amplicon microbial surveys. Bacterial diversity in these areas tends to be dominated by organisms within the *Proteobacteria* and *Bacteroidetes* phyla, with members of *Methanosaeta*, *Methanolobus* and *Clostridium* also present [29, 30].

To study the diversity of hydrogen-producing bacteria and methanogens in China using the metagenome sequencing method, fresh coal samples were selected from several regions. The *Illumina Miseq* sequencing technique was used to determine microorganisms in these coal samples; and the diversity and structure of microorganisms were analyzed according to the mine geological information of sampling areas and environmental conditions in the coal seam. From a biological perspective, the systematic study of the species composition and abundance of microorganisms in the coal seam has a positive effect on the enrichment of the biological content in this field; the research thus provides a theoretical basis for microbially enhanced CBM (MECBM). For the lower permeability and the unworkable coal seam, local microorganisms produce biogenic gas, which can increase the yield of CBM to assure the best use of resources. We propose that the mechanism of carbon dioxide reduction could be better understood by studying the metabolic pathways of these bacteria in situ in the coal seam and that once the technology capable of introducing carbon dioxide into the coal seam to produce methane has been established, this knowledge can be used in turn to reduce emissions; as well as the greenhouse effect. In addition, it is of significance to determine the dominant bacteria and general species abundance of hydrogen-producing bacteria and methanogens in samples from different geographical regions.

## Results

### Sequencing results and bacterial diversity index

Using the Illumina MiSeq system, 514,633 filtered bacterial gene sequences were obtained from 10 samples, with each sample producing 51,466.3 sequences on average (range 40,470–62,999; SD = 7883.13). Additionally, 593,084 filtered archaeal gene sequences were obtained from 10 samples, with each producing 58,308.4 sequences on average (range 17,838–94,080; SD = 26,756.6). A total of 24 archaeal and 250 bacterial genera were detected from all sequences. The species richness and diversity, including ACE, Chao1, Shannon and Simpson index, are showed in Table 1. OTUs containing only one sequence were discarded.

**Table 1:** The analysis of bacterial diversity index in coal samples

Coal no.	Seq num	OTU num	ACE index	Chao1 index	Shannon index	Coverage (%)	Simpson
C1	39,766	91	115.83	104.32	1.98	98	0.26
C2	41,667	227	261.37	240.86	1.38	100	0.40
C3	46,582	436	498.10	474.17	3.18	100	0.09
C4	46,471	535	159.95	148.27	1.52	100	0.40
C5	52,593	196	207.55	200.12	2.22	100	0.16
C6	59,965	443	503.16	472.52	1.56	100	0.46
C7	59,986	417	502.79	458.36	1.98	96	0.24
C8	55,497	504	524.73	510.44	2.61	94	0.14
C9	44,387	166	185.59	176.33	1.28	98	0.42
C10	46,216	208	259.89	237.06	1.58	98	0.34

The Shannon index and the Simpson index are two measures of microorganism diversity. The larger the Shannon index, the higher the diversity of the community. However, the larger the Simpson index, the lower the diversity of the community [31]. The Shannon index is the smallest and the Simpson index is the largest in C4, indicating that C4 has the lowest community diversity and C3 has the highest community diversity. The species abundance in C3, C6, C7 and C8 was relatively higher than other samples (Table 1).

**Fig. 1:**
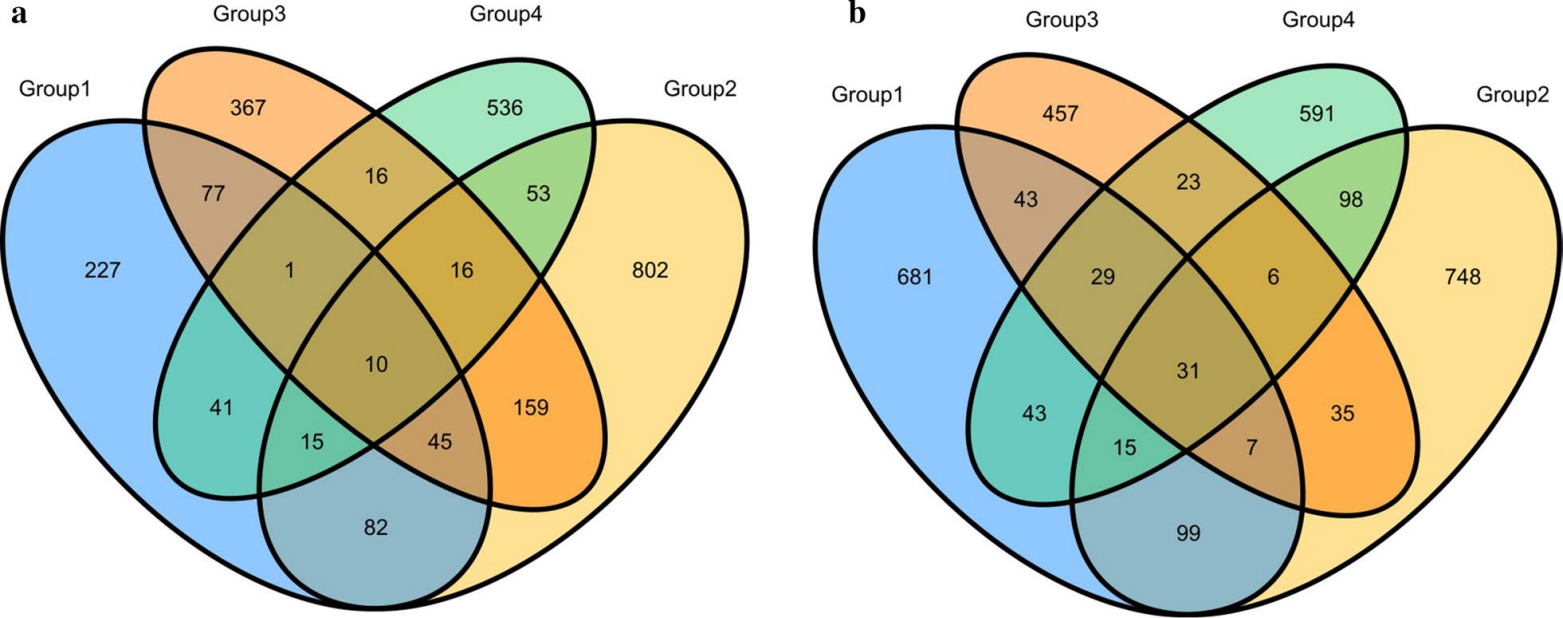
The Venn diagram showing the number of shared and unique OTUs between different Groups for bacterial community (**a**) and archaea community (**b**). Group 1: C1, C7; Group 2: C2, C3, C6, C10; Group 3: C4, C9; Group 4: C5, C8

To discern the difference between the samples and OTU abundance, a four-way Venn diagram was conducted (see Fig. 1). Coal samples were divided into four Groups according to the coal rank—the value of *R*_O_ is commonly used to evaluate the coal rank in coal geology [32]. Note that the higher the *R*_O_ value, the higher the coal rank. Group 1 included low rank bituminous coal samples (C1, C7), Group 2 consists of medium rank bituminous coal samples (C2, C3, C6, C10), Group 3 included the high coal rank bituminous coal samples (C4, C9), and Group 4 consists anthracite (C5, C8). Ten OTUs were found to be common to the four groups in the bacterial community, there were 31 OTUs shared in the archaea community. The samples from Group 2 (0.8% < *R*_O_< 1.1%) and Group 4 (2.67% < *R*_O_< 3.15%) showed a higher number of OTUs than those from other Groups based on the observed bacterial OTUs. The highest number of unique OTUs was observed in Group 2 (0.8% < *R*_O_< 1.1%) and the lowest was found in Group 1 (*R*_O_< 0.6%). These results showed that a relatively high number of unique OTUs in Group 2 and Group 3 (1.4% < *R*_O_< 1.8%) for archaea community (Fig. 1b), indicating that the archaea community was more susceptible to coal rank than the bacterial community; and that the number of OTUs in low and medium rank coal are higher than those of high rank coal.

**Fig. 2:**
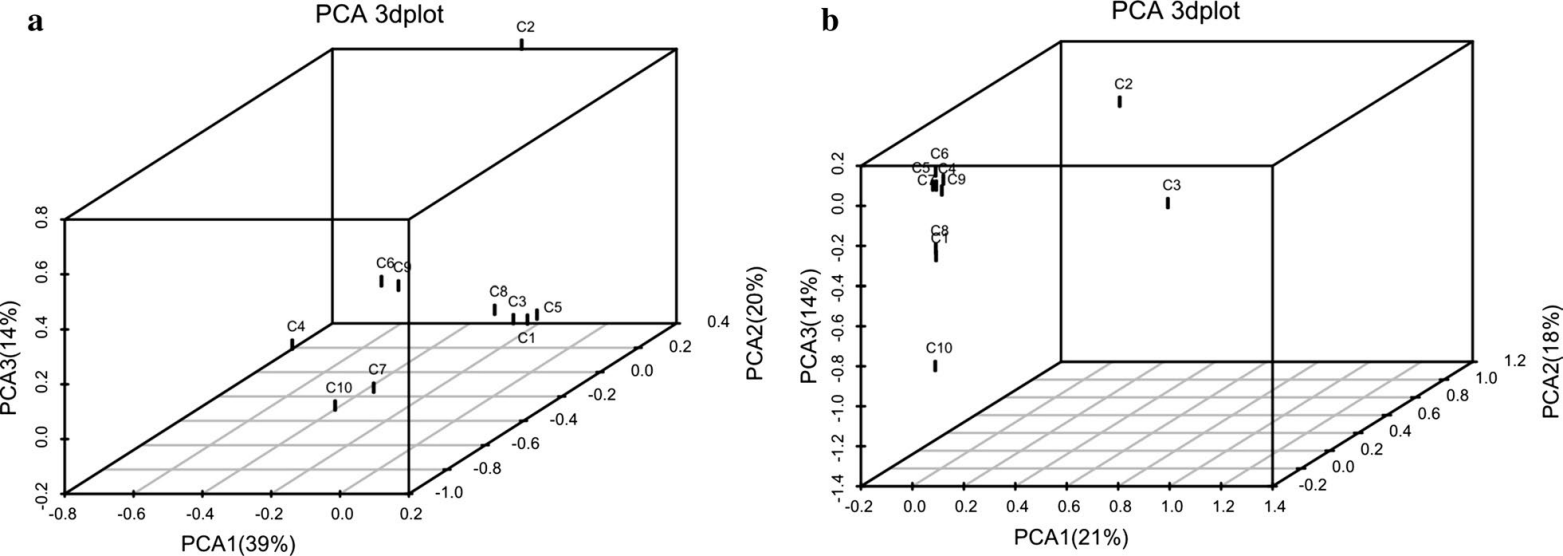
Principal component analysis (PCA) base on OTUs for bacterial community (**a**) and archaeal community (**b**). The higher the degree of similarity between samples, the more aggregated in the graph

The PCA analysis results suggested that an obvious separation of the communities in different samples (see Fig. 2). The first and second axes of this figure showed values of cumulative percentage variance of species equal to 39% and 20%, respectively. In total, 59% variances of species were explained by the two axes in Fig. 2a; and 39% variances of species were explained by the two axes in Fig. 2b. The PCA analysis indicated that the bacterial communities were more diffuse than archaeal community overall. The bacterial communities were similar between C6 and C9. As a result, sample C1, C3, C5, and C8 clustered together, and sample C2 was clearly separated from the other samples (Fig. 2a). However, the distance between the samples is closer except C2 and C3 in the archaeal community (Fig. 2b), indicating the bacterial community are more susceptible to regional influence than archaeal community.

### Bacterial community structure

**Fig. 3:**
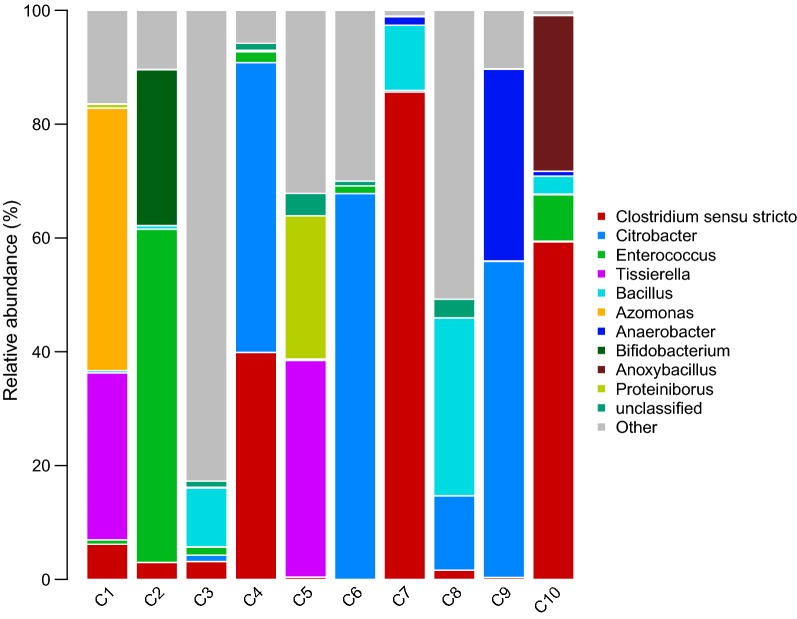
Genus level taxonomic composition of the top 10 most abundant genus from 10 coal samples for the bacterial community. Total summed abundances of the remaining genus are indicated by the “other” group and unclassified

**Fig. 4:**
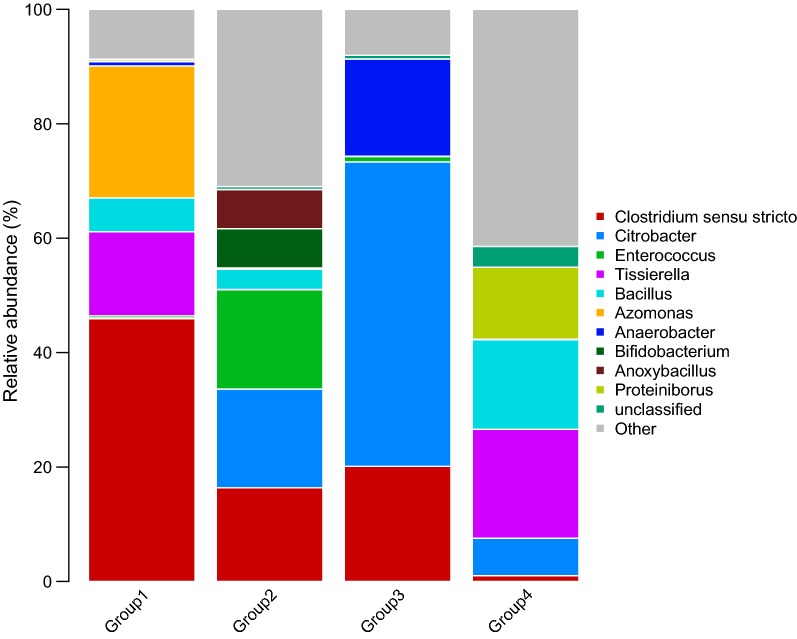
Genus level taxonomic composition of the top 10 most abundant genus from four Groups for the bacterial community. Total summed abundances of the remaining genus are indicated by the “other” group and unclassified

Members of phyla *Firmicutes*, *Proteobacteria*, *Bacteroidetes*, *Actinobacteria*, *Acidobacteria*, *Verrucomicrobia*, *Planctomycetes*, *Chloroflexi*, *Nitrospirae* were detected in 10 areas after high-throughput sequencing. The dominant phyla detected in these Groups were *Firmicutes* and *Proteobacteria*; *Actinobacteria* has a large proportion in Group 3. Although the microorganisms for each of the group samples were mostly dominated by membership in the families *Clostridiaceae*, *Bacillus*, *Enterobacteriaceae*, and *Clostridium,* there are differences at the genus level that are shown in Fig. 3. We find the diversity of bacteria in biogenic CBM areas, such as C1, C2, C3, C4, are higher than in the areas without biogenic in C8, C9, C10. The genus *Clostridium senus stricto* was detected in Groups and was the highest abundance genus occurring in Group 1. *Clostridium senus stricto* (45.92%), *Tissierella* (14.68%) and *Azomonas* (23.08%) were the dominant genera in Group 1. Group 2 was dominated by *Clostridium senus stricto* (16.37%), *Citrobacter* (17.26%) *and Enterococcus* (17.36%). The most abundant members of Group 3, ranking in the top three, were *Citrobacter* (53.22%), *Clostridium* sensu stricto (20.12%), and *Anaerobacter* (16.96%). The bacterial community in Group 4 (see Fig. 4) was mainly composed of *Tissierella* (19.02%), *Bacillus* (15.66%), *Proteiniborus* (12.59%), and *Citrobacter* (6.53%).

It is worth noting that traces of acetogens can be detected in the coal seam of each region, which is strictly an anaerobic or facultatively anaerobic group of bacteria. The acetogens use the products of primary fermentation, such as propionic acid, butyric acid, lactic acid and other substances to produce acetic acid and H_2_. This work shows that acetogens have a narrow ecological niche and they are more likely than methanogens to be affected by changes in the external environment [33], particularly changes in temperature and pH value, etc. To maximize the production of hydrogen, the genera of acetogens in the coal seam should be detected accurately and optimum growth conditions ensured. Traces of *Bacillus* and *Clostridium* were determined in all areas except C4. *Bacillus* can produce a kind of fatty peptide biosurfactant, which can enhance the thermal stability of cellulases to promote lignocellulose degradation. The resulting hydrolysate can serve as a substrate for cellulolytic *Clostridium* [34]. In addition, *Exiguobacterium* in C1, C6 and *Pseudomonas* in C3 could produce extracellular cellulases, which break down various types of biomass to provide the substrate for the next stage of fermentation [35, 36]. Both *Clostridium* and *Enterobacter* are potential hydrogen-producing contributors in the acetogenesis stage of hydrogen production [37]. For example, *Acetanaerobacterium* in C1 can degrade carbohydrates [38]. *Acinetobacter* in C8 can degrade a variety of aromatic compounds [39]. The *Klebsiella* sp., detected in C4, C6, and C9 have the unique ability to degrade polycyclic aromatic hydrocarbons, carbazole, and other substances [40–43]. There is evidence that *Pseudomonas* and *Citrobacter* can also participate in redox reactions, which were reported to co-operate with the hydrogen-producing bacteria to degrade polycyclic aromatic hydrocarbons and toluene into H_2_, CO_2_ and acetate. The literature also shows that hydrogenase is present in bacteria such as *Enterobacter*, *Clostridium*, and *Klebsiella*. These microorganisms are also potential contributors to the production of hydrogen [44, 45].

*Hydrogenophaga* in C10 is a typical aerobic bacterium that uses oxygen to oxidize hydrogen [46] and should, therefore, not be detected in an anaerobic coal seam; however, this bacterium may be capable of anaerobic metabolism. The SRB in C1, C3 and C7 were located in the subsurface environment and involved in the recycling of sulfur and carbon when in the presence of sufficient carbon sources and energy substances that can be metabolized to produce H_2_S. This system can combine with ferrous ions to produce insoluble iron sulfide [47, 48]. The *Rhizobium* detected in C7 is generally found in the roots of leguminous plants; as well as in anaerobic activated sludge [49], which may be a type of bacteria involved in the nitrogen cycle.

### Archaeal community diversity

The archaea were classified into phyla *Euryarchaeota*, *Thaumarchaeota*, *Woesearchaeota*, and *Pacearchaeota* with the high-throughput sequencing method. The *Euryarchaeota* were identified as the dominant bacterial phyla. *Methanomicrobia* and *Methanobacteria* were also identified to class level. The methanogens belonging to the phyla of *Euryarchaeota* have strong substrate specificity and can only use simple organic matter with no more than two carbon atoms. According to the different metabolites, methanogens were divided into three types: the (1) hydrogenotrophic methanogens that can use H_2_ and CO_2_ to produce methane; the (2) methylotrophic methanogens, which can use methyl compounds, such as methanol, methylamine, and formic acid, and (3) the methanogen which can use of acetic acid, termed acetoclastic methanogens [50–52].

**Fig. 5:**
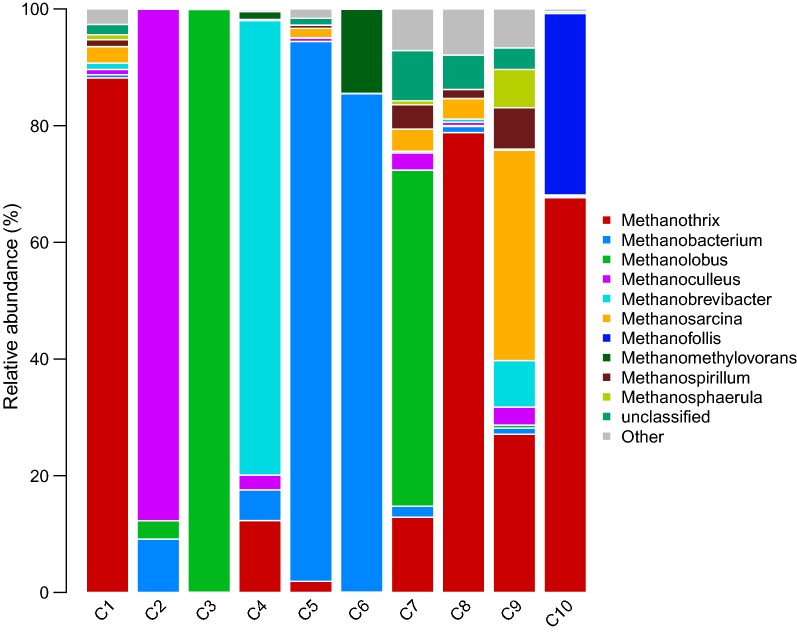
Genus level taxonomic composition of the top 10 most abundant genus from 10 coal samples for the archaeal community. Total summed abundances of the remaining genus are indicated by the “other” group and unclassified

**Fig. 6:**
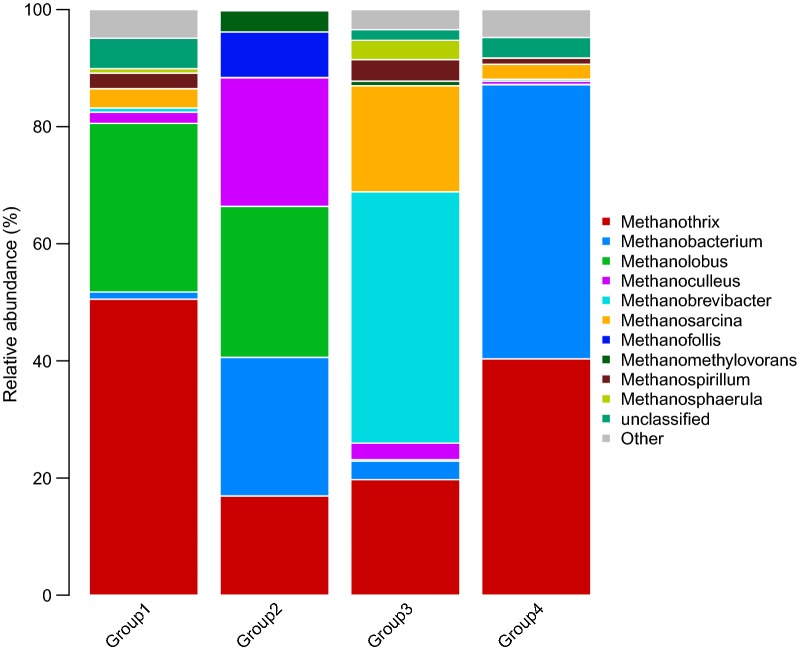
Genus level taxonomic composition of the top 10 most abundant genus from four Groups for the archaeal community. Total summed abundances of the remaining genus are indicated by the “other” group and unclassified

At the family level, the predominant archaea in Group 1 were *Methanotrichaceae* and *Methanosarcina.* The dominant families in Group 2 were *Methanosarcinaceae* and *Methanomicrobiaceae*. *Methanotrichaceae* was the dominant family in Group 3. In Group 4, they were *Methanotrichaceae* and *Methanomicrobiaceae*. Data shown in Fig. 5 reflects the differences of methanogens at genus level. As shown in Fig. 6, the Group 1 was dominated by *Methanothrix* (50.24%) *Methanolobus* (28.8%), and *Methanosarcina* (3.3%). Group 2 was dominated by *Methanoculleus* (21.95%), *Methanolobus* (25.8%), *Methanobacterium* (23.65%), and *Methanothrix* (16.94%). The dominant methanogens in Group 3 were *Methanobrevibacter (42.92%)*, *Methanothrix* (19.72%), *Methanobacterium* (3,14%), and *Methanosarcina* (18.12%). *Methanobacterium* (46.79%) and *Methanothrix* (40.35%) dominated Group 4. Group 2 and Group 3 have a diverse composition in methanogens. There are also traces of hydrogenotrophic methanogens in these areas. Hydrogen partial pressure plays an important role in the last methanogenic stage, and thus hydrogen-consuming bacteria appear to be particularly important. The hydrogenotrophic methanogens detected in this study are the main consumers of H_2_ in the fermentation stage. The methanogens associated with *Methanoculleus* in C2, *Methanobrevibacter* in C4, *Methanobacterium* in C5 and C6, and *Methanosarcina* in C9 are all hydrogenotrophic methanogens that reduce the hydrogen partial pressure in the system through interspecific hydrogen transfer to promotes acetogenesis.

## Discussion

### Analysis of factors affecting the microbial community structure

**Fig. 7:**
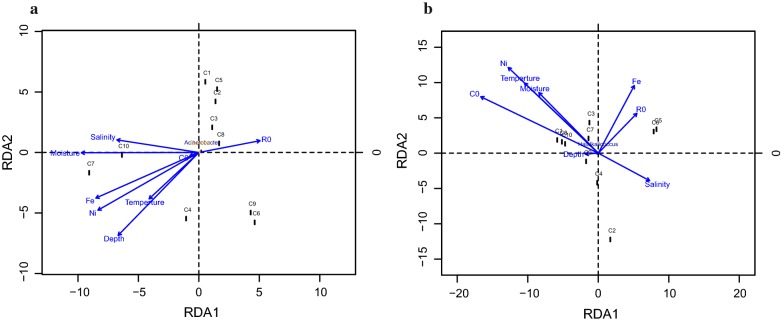
The RDA (redundancy analysis) based on the level of bacteria (**a**) and archaea (**b**) with the coal bed environmental factors and coal characteristics. The length of the impact factor is longer, the contribution of the impact is higher, and conversely, when the impact factor is shorter, the contribution of the impact is lighter. When the environmental factor is acutely angled with the sample, there is a positive correlation, and when the environmental factor and the sample angle are obtuse, there is a negative correlation

To further investigate possible relationships between the environment factors and community variance, RDA analysis was created (Fig. 7). In total, eight environment factors, including trace elements Fe, Co, Ni, temperature, salinity, depth, moisture, and *R*_O_. The depth and reservoir temperature were measured in the sampling location and other information were obtained from the geological data of local mines (see Table 2). Data shown in Fig. 7a revealed that the bacterial community compositions found in this study were significantly affected by Fe, Ni, moisture, salinity, and *R*_O_. All communities, other than C4, C7, C10, are positively correlated with *R*_O_; C4, C7, and C10 positively correlated with Fe, Ni and moisture. Co is required for co-enzyme M methyl-transferase, which is an important enzyme in the biochemical metabolism of methanogens [53]; therefore, the effect of Co on archaeal community is greater than that of bacterial community. The elements Fe, Co, and Ni; as well as moisture, appeared to be the most significant environmental factors followed by *R*_O_ and salinity in the archaeal community. There is a significant positive correlation between Co and the C1, C8, C9, C10 communities. All communities except C2, C4, C6, and C5 were negatively correlated with salinity (Fig. 7b).

**Table 2:** The environment information and coal samples

Coal no.	Location	Depth (m)	Temperature (°C)	Salinity (g/L)
C1	Yima	500	24.90	0.75
C2	Liyazhuang	578	26.30	0.80
C3	Shaqu	557	26.80	0.50
C4	Hebi	580	25.40	1.00
C5	Jingcheng	550	25.90	0.90
C6	Suzhou	590	26.50	0.70
C7	Neimeng	880	26.00	1.25
C8	Jiaozuo	465	27.20	0.85
C9	Shoushan	820	34.60	0.33
C10	Pingdingshan	670	40.10	0.50

### Coal rank

**Fig. 8:**
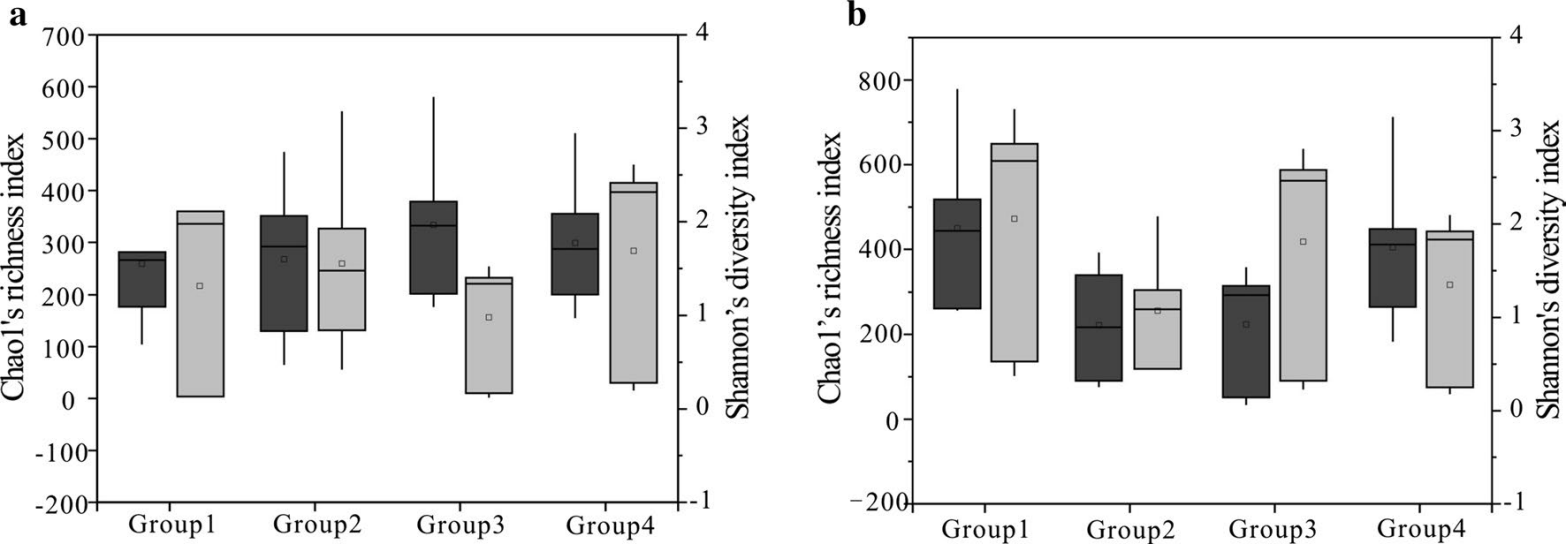
Chao1′s (dark grey) and Shannon’s (light grey) index for the four groups (Coal samples were divided into four groups according to the value of RO for the bacterial community (**a**) and archaea community (**b**), Group 1 represents a value less than 0.6%, Group 2 represents the value between 0.8 and 1.1%, Group 3 represents the value between 1.4 and 1.8%, Group 4 represents the value between 2.67 and 3.15%) derived from regions. 25th and 75th percentiles are indicated by the outer edges of boxes while the maximum and minimum values are showed by the ends of the whiskers and the median by a horizontal line within each box

The coalification “jump” refers to a series of physical and chemical changes under the temperature and pressure of coal during geological history. Coal has thus undergone a process of gradual to sudden change. The four jumps correspond to *R*_O_ of 0.6, 1.3, 2.5, and 3.0%. Regardless of the archaeal or the bacterial community under consideration, the coal rank has a certain influence on the diversity and abundance of the bacteria. With an increase of coal rank, in both the archaeal and the bacterial communities, the diversity of community shows a certain downward trend overall (Fig. 8). Moreover, microorganisms may impact the composition of the coal controlled by the coal ranks. The middle and low rank coals contain large amounts of plant evolved substances in Group 1, which contains a lot of plant evolved substances. Here, there is higher content of hydrogen, oxygen, and nitrogen; and the nutrients required by the bacteria are abundant. In the process of coalification, organic substances generate a lot of moisture and liquid hydrocarbons. At the same time, the side chains of hydrogen and oxygen contained in coal are also abundant. These liquid and solid substances provide the foundation of life for bacteria. As a result, the abundance and diversity of hydrogen-producing bacteria and methanogens in coal in this region are relatively high. With the increase of *R*o, the side chain content of hydrogen and oxygen in coal is drastically reduced and the components available to the microorganisms are also reduced. Therefore, the species abundance and diversity of the bacterial and archaeal communities in Group 2 and Group 3 are reduced overall. So far, the coal ranks of biogenic coalbed methane have been found in Nature to have a reflectivity of 2.0% (C4 Hebi). After *R*_O_ > 2.5%, the organic compounds that can be converted to small molecules have been very rare, but there has been a higher diversity and abundance in Group 4. We speculate that the nutrients introduced by groundwater at this time are available for bacterial reproduction. The nutrient components brought about by groundwater in different regions and in different seasons may have contributed to diversity of species. One reason for the higher flora diversity in Group 4 may be that C8 Jiaozuo Jiulishan area has better groundwater runoff conditions and stronger recharge. It can transport nutrients for the flora, so the diversity and abundance are higher than Group 2. It is worth noting that the diversity and abundance of the archaeal communities are negatively correlated with coal ranks to a certain extent. However, the species abundance in the bacterial communities is positively correlated with coal ranks, and the diversity shows a downward trend. With the rise of coal ranks, some bacterial groups gradually adapted to the environment of various coal ranks and can grow and multiply in large numbers, and methanogens are difficult to adapt to coal ranks.

### Trace metal elements

Trace metal elements can promote the growth of microorganisms within a certain range, where the cell maintains homeostasis of the elements through metabolic regulation. Trace metal elements can also exist in various enzymes, which can be absorbed and used by microorganisms in the process of anaerobic metabolism, which has an influence on the community structure of hydrogen-producing bacteria and methanogens (Table 3).

**Table 3:** The role of Fe, Co and Ni in reaction and transformation in anaerobic metabolism [61, 62]

	Element functions		Element functions		Element functions
Fe	Hydrogenase CO-dehydrogenase Methane monooxygenase NO-reductase Superoxide dismutase Nitrite and Nitrate reductase Nitrogenase	Ni	CO-dehydrogenase Acetyl-CoA synthase Methyl-CoM reductase (F430) Urease Stabilize DNA, RNA Hydrogenase	Co	B12-enzymes CO-dehydrogenase Methyltransferase

**Fig. 9:**
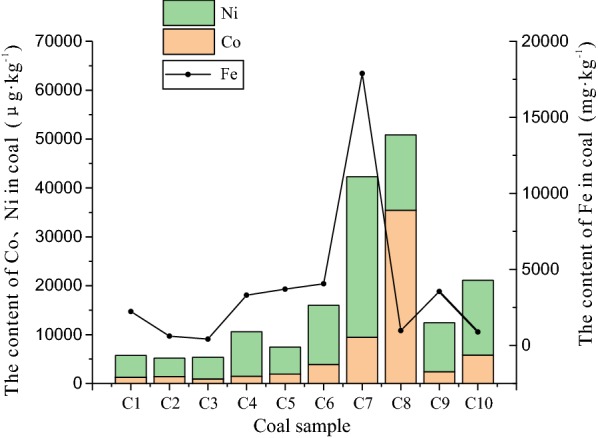
The trace metal elements content of Fe, Co, Ni in coal samples

Fe and Ni have a greater effect on hydrogen-producing bacteria than Co [54–56]. Fe and Ni can participate in the synthesis and metabolism of hydrogenases and other metalloenzymes in microorganisms. As the content of Fe and Ni increases within an certain range, so does the abundance and diversity of hydrogen-producing bacterial populations. The content of Fe and Ni in C7 is much higher than that in other regions, and this work has found that *Clostridium* are hydrogen-producing bacteria. This finding indicates that excessive levels of the trace elements may have a toxic effect on the growth of microorganisms and inhibit the activity of metalloenzymes. Levels of Fe in C4, C6, and C9 were not significantly different and were stable at 3500 mg Kg^−1^ (Fig. 9). The relative abundance of Ni in the three areas is C6 > C9 > C4, which corresponds to abundance order (also C6 > C9 > C4), but the order of diversity is C6 > C4 > C9. Members of genera: *Clostridium*, *Klebsiella*, *Enterobacter* and *Citrobacter* were detected in the C4, C6 and C9 communities; including those at higher abundances and levels of diversity than from other regions.

In the archaeal community, the influence of Fe, Co, and Ni on the methanogens is even more important. Co is a key element in the synthesis of methanogenic coenzyme F_430_ [57], and the Co content in the top three is C8 > C7 > C10, methanogen species and abundance being C8 > C7 > C10. Content of Co is positively correlated to the abundance and diversity of methanogens to a certain extent. Although the content of Fe in C7 is much higher than that of other regions, it does not affect the distribution of methanogens in the region. There are only few types of methanogens that may contain Fe—in previous studies, only one species, named *Methanothermobacter*, was discovered. The presence of monoferric hydrogenase in methanogens of *M. marburgensis* catalyzes the reversible reaction of methenyl-H_4_MPT^+^ and H_2_ to generate methylene-H_4_MPT and H^+^; producing methane from CO_2_ and H_2_ [58]. Methanogens using hydrogenotrophic metabolism may also contain similar enzymes. In addition, a large proportion of methylotrophic methanogens are also speculated of harboring such enzymes except the *Methanoculleus* and *Methanobacteria.* It is speculated that there may be a metalloenzyme associated with Fe in the methylotrophic methanogens.

### Groundwater conditions

Groundwater directly or indirectly provides an ecological basis for the growth and metabolism of extremophiles in the coal seam. On the one hand, groundwater recharge supplies large amounts of nutrients for bacterial and archaeal communities; on the other hand, groundwater environmental conditions (Eh, pH, salinity, ion composition, and trace elements) directly affect microbial growth and metabolic enzyme activity. Groundwater environmental conditions are directly related to the use and degradation of coal, and the microorganisms located in the coal seam show different community structures and functional characteristics.

Microbial nutrient substrates are generally dissolved. The runoff zone in the mining area can allows the survival of coal seams. High permeability reservoirs have a positive impact on the growth and reproduction of hydrogen-producing bacteria and methanogens, whereas metamorphism has a significant negative impact on coal permeability in coal reservoirs [59, 60]. In areas with biogenic CBM, the C2, C4, C6 and C7 communities have all been well documented. These communities belong to the low and medium coal rank, the porosity of coal is relatively higher than high rank coal, groundwater can provide nutrients to the microbes in the coal seam in time. The current CBM development zone within the Powder River Basin in the U.S. is mainly concentrated in the groundwater runoff zone. The gas stable isotope data from a shallow CBM well in the C6 mining area also confirmed the presence of biogenetic CBM in the area. However, gas stable isotope data from another deep CBM well indicated that the CBM is mainly thermogenic. These results show that as the depth of burial increases, the runoff conditions will weaken and it will be difficult to transport nutrients for the microorganism, which will result in a decrease in the abundance and diversity of the community. The roof and floor of the No. 2 coal seam in C2 area have relatively stable layers of mudstone and clay rock, which makes it difficult for the hydrogen-producing bacteria and methanogens in the coal seam to obtain liquid nutrients, and limits their growth and metabolism and, therefore, their community diversity and abundance. Note that in this area the Chao1 index is 240 and the Shannon index is 1.38 in the bacteria community. The Chao1 index of methanogens is 82, the Shannon index is 0.56. The sandstone fissured aquifer roof in C4 area of the No. 2_1_ coal seam has better recharge conditions and fills the coal seam with water. It is possible that the microbial community experiences cumulative effects from the sufficient availability of different nutrients, which affects transportation, compared with the abundance and diversity of the microorganism community in the C2 area, which has greatly improved. In this area, the Chao1 index of hydrogen-producing bacteria is 148, the Shannon index is 1.52; the Chao1 index of methanogens is 368, and the Shannon index is 2.35. The sandstone fissured aquifer of C6 area is a direct water-filled aquifer of No. 3 coal seam. Fracture development within the layer and moderate aqueosity also plays an active role in the abundance and diversity of community. Here, the Chao1 index of hydrogen-producing bacteria is 472, the Shannon index is 1.56; the Chao1 index of methanogens is 384 and the Shannon index is 1.08. This is also the case in the C7 area, the No.5 coal seam has a directly fractured aquifer with good recharge conditions, the abundance index of the hydrogen-producing bacteria is 458, the Shannon index is 1.98; and the Chao1 index of the methanogens is 256 and the Shannon index is 2.47. Therefore, the species diversity of hydrogen-producing bacteria and methanogens in C4, C6, and C7 is higher than that in C2.

Groundwater environmental conditions will directly affect the growth and metabolism of microorganisms. The pH value of coal-bed groundwater is generally neutral, but the pH value varies between 6.5 and 8.4 in sandstone fractured aquifer in C4 area No. 2_1_ coal seam and the salinity is 1.0 g L^−1^. In the direct aquifer layer of the No. 3 coal seam in the C6 area, the pH ranges between pH 6.8 to 8.0, and the salinity is 0.7 g L^−1^. The groundwater pH value of the C7 area is 6.1–7.3, and the salinity is 1.25 g L^−1^. The pH value in C4, C6, and C7 is close to neutral and the degree of mineralization is low, where the microorganism community has better growth, higher abundance and higher diversity. In addition, the groundwater salinity and ion composition are closely related to the anaerobic reduction environment of the coal seam. For example, SO_4_^2−^ is used to evaluate the closed conditions of groundwater, and HCO_3_^−^ is the product of the anaerobic desulfurization reaction of SO_4_^2−^, so high HCO_3_^−^ can be used as a sign of good sealing and strong reduction of coal-bed groundwater [63]. The water chemistry in the C4 area is HCO_3_·SO_4_–Ca·Mg, the water chemistry in the C7 area is similar to the C4 area, HCO_3_·SO_4_–Ca·Na, and provides a relatively closed anaerobic environment. In this case, the Chao1 index of hydrogen-producing bacteria in C4 is 148, the Shannon index is 1.52; the Chao1 index of methanogens is 368, the Shannon index is 2.35. The Chao1 index of hydrogen-producing bacteria in C7 is 458, the Shannon index is 1.98, the Chao1 index is 256, and the Shannon index is 2.47. In C6, the water chemistry is SO_4_·HCO_3_–K·Na, and SO_4_^2−^ is dominant, whereas the Chao1 index of hydrogen-producing bacteria in C6 is 472, the Shannon index is 1.56, the Chao1 index of methanogens is 384, and the Shannon index is 1.08. Data show that diversity in C6 is slightly lower than C4 and C7.

Some hydrogen-producing bacteria and methanogens were detected in C8 and C9 areas in the area where no biomethane was found. It was also noteworthy that the groundwater conditions of these two areas are similar to those in the above-mentioned biogenic methane areas, which are located in the groundwater runoff zone and the groundwater recharge is more able to transport some organic matter into the coal seam, so that a large number of bacteria grow and multiply, which is one of the reasons for the higher species abundance and diversity of C8 and C9.

### Temperature

Temperature and trace metal elements influence the abundance and diversity of microbial communities by directly changing both the growth and metabolism of microorganisms and their metabolic environment. Thus, from a microbiological point-of-view, optimum temperature is one of the most important factors that influences microorganism growth and metabolism. Figure 5b show that temperature exerts a relatively weak influence on the abundance and diversity of methanogens, even though hydrogen-producing bacteria exist within a narrow ecological amplitude and are sensitive to temperature change. This variable is correlated with species abundance and diversity, and the results of this study show that the temperature of the coal seam (i.e., between 25 and 27 °C) is positively correlated with bacterial population abundance. At C8, the temperature was 27.2 °C, the highest temperature recorded in this study. The Chao1 index of hydrogen-producing bacteria was 510 and the Shannon index was 2.61, also the highest among samples (C1–C8). The lowest temperature, 24.9 °C, was found at C1, where the Chao1 index of bacterial community was the lowest. The abundance and diversity of microbial species increases with temperature in C3 > C6 > C2 > C7 > C5 > C4. Geothermal gradient anomalies at C9 and C10 caused much higher temperatures; the ambient temperatures at C9 and C10 were 34.60 °C, and 40.10 °C, respectively. The Chao1 index of hydrogen-producing bacteria in C9 was 176, and the Shannon index was 1.28; the Chao1 index is 237, Shannon’s index is 1.58. Compared to the first eight areas, abundance and diversity has slightly decreased. Here, both hydrogen-producing bacteria and methanogens can grow and reproduce at the ambient temperature.

### Microbial syntrophic interactions

In the extreme environment of the coal seam, consortia of bacteria are formed among the microorganisms in the coal seam. Through the exchange of metabolites and micro-environmentally controlled symbiosis, competition and resource allocations maintain the specific functions of the microbial community, which determines the biomethane production pathway in the coal seam. *Methanothrix,* which convert acetic acid into methane, is the dominant genus in the methanogen community of the C1 area. The bacteria associated with *Alkalibaculum* and *Desulfosporosinus* are homoacetogenic bacteria that use H_2_ as the electron donor to produce acetic acid. They are the main competitors for hydrogenotrophic methanogens and also provide metabolic substrate for methanogens. Hydrogen-producing bacteria such as *Clostridium* and *Tissierella* also provide acetic acid, and thus the high abundance of hydrogen-producing bacteria provides a rich metabolic substrate for *Methanothrix*. Together, the methanogens and the hydrogen-producing bacteria are in syntrophic interaction and the methane generation pathway in this area is determined by the decomposition of acetic acid. The methanogens in C2, C4, and C6 are mainly hydrogenotrophic methanogens. Hydrolytic fermentation bacteria and acetogens both contribute to the production of acetic acid and H_2_. They also produce enzymes, cofactors and metabolic signals to regulate the hydrogen production. Furthermore, homoacetogenic bacteria and acetogens do not compete in these areas. Hydrogenotrophic methanogens can produce methane from CO_2_ and H_2_ produced in the previous stage. Therefore, metabolic pathways in these areas are mainly used for H_2_, formate, and other substances.

More than 99% of the C3 area harbors methylotrophic methanogens, such as: *Methanolobus*. *Brevibacter*, *Paenibacillu*s, *Brochothrix*, and *Lactococcus*. Previous studies showed that methoxyaromatic compounds (an important part of lignocellulose), are degraded to produce methanol and other substances [64]. Microorganisms in this region may degrade the lignocellulose-like matter of coal to provide resources to methylotrophic methanogens. This simple microbial community cannot provide sufficient substrates for methanogens that consume H_2_. The biomethane production pathway in this area is based on the consumption of methyl compounds.

*Staphylococcu*s was also detected in the C3 area. Recently, *Staphylococcus AntiMn*-*1* was isolated from deep-sea sediments in the Clarion-Clipperton area with high manganese content. It contained genes with high resistance to manganese, which is thought to be an adaptation to the marine sedimentary environment [65]. The heavy metal content in the C3 area is relatively high. It may be that the coal seam environment can effectively induce the expression of resistance genes, which may have antagonistic and detoxifying effects on the transport and toxicity of heavy metals within microorganisms. *Staphylococcus* in this area may contain resistance genes to adapt to the coal seam environment so that it may also participate in the fermentation metabolism of coal. There are many different species of methanogens in C7, and the hydrogen-producing bacteria is dominated by *Clostridium*, *Bacillus*, *Citrobacter*, and other anaerobes, which provide substrates for acetoclastic methanogens and also H_2_, CO_2_, and formate for hydrogenotrophic methanogens. Furthermore, accumulating acetic acid reduces sulfate-reducing bacteria, including *Desulfosporosinus* and *Desulfitobacterium*. The SRB have a stronger affinity for acetic acid than acetoclastic methanogens, but they do not compete with methylotrophic methanogens for certain substrates, such as methanol. Thus, the metabolisms of both sulfate-reducing bacteria and methanogens can proceed simultaneously in this area [66]. Metabolism in C7 was dominated by methylotrophic methanogens, followed by acetic acid fermentation and then carbon dioxide reduction.

## Conclusion

The data presented in this study reveals a level of in situ hydrogen-producing bacteria and methanogen diversity within the coal seam. Indeed, in some areas of biogenic CBM, microbial consortia consist of coal microorganisms to such an extent that two, or more, microbial groups with complementary metabolic activity comprise these specific systems. Thus, through the exchange of metabolic substrates and microenvironmental regulation symbiosis, groups compete with one another for resources to generate biological methane. In contrast, in areas where biogenic CBM is absent, traces of hydrogen-producing bacteria and methanogens were detected, and it is suspected that the geological and environmental conditions in these regions were unable to provide optimal growth environments for microbial communities involved in metabolic processes.

The diversity and abundance of in situ hydrogen-producing bacteria and methanogens within the coal seam studied in this paper are influenced by both biological and non-biological factors. The influence of coal rank on species abundance and diversity is such that microorganisms can grow even in high rank samples to some extent, where the bacterial community is more diverse than the archaeal community. Good groundwater conditions are also more likely to affect the growth of methanogens than is coal rank, as seen in the Jiaozuo (C8) sample. Coal bed temperatures between 25 and 27 °C are the most conducive for the growth of hydrogen and methane-producing bacteria and lead to higher species abundances. The presence of the elements Fe, Co, and Ni also promotes the growth and metabolism of both hydrogen and methane-producing bacteria, whereas mutual competition and promotion amongst microbial communities are also key factors influencing community distribution characteristics. The interactions between these variables also determine the fermentation and metabolism pathways of methanogens in the coal seam.

## Methods

### Samples and conditions of collection

**Fig. 10:**
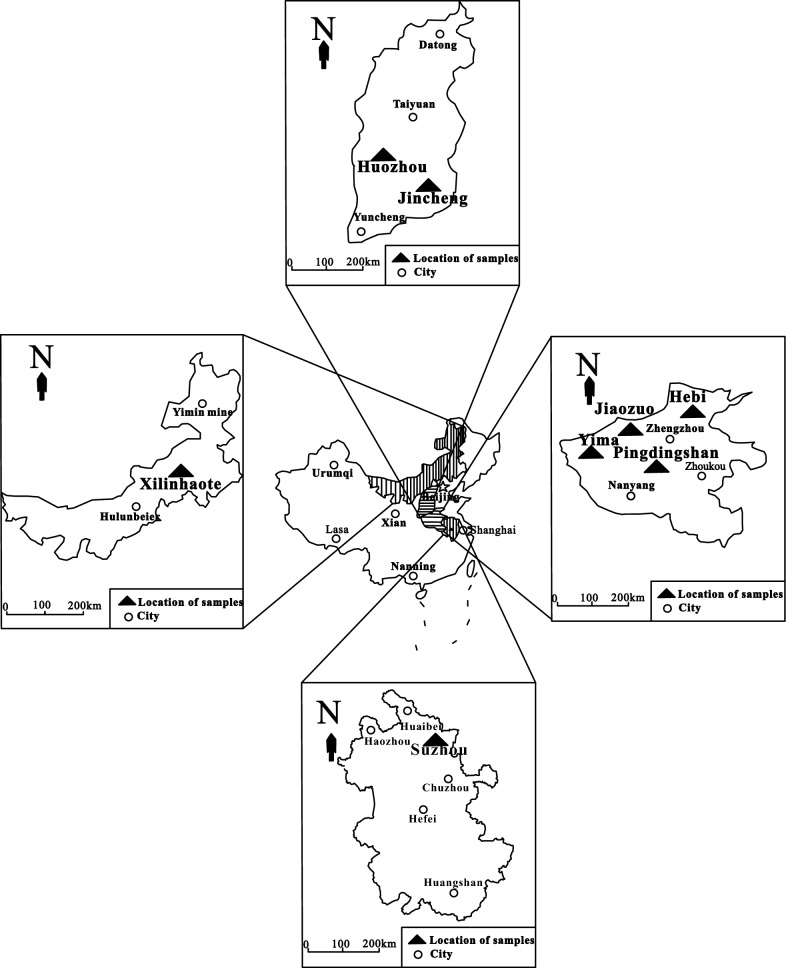
The location of coal samples

The coal samples used in this experiment were collected from different regions of China (Fig. 10). Fresh coal samples were taken from the coal mine working face and placed in a sterilized, low-temperature anaerobic tank and sent to the biological laboratory for storage at − 20 °C. At the same time, the environmental conditions and basic information such as depth of burial, temperature and degree of groundwater mineralization are collected (Table 3).

### Coal quality characteristics

Coal proximate analysis, ultimate analysis and *R*_O_ values are shown in Table 4. Table 4 shows that the experimental samples can be sorted into grades, such as low rank bituminous coal (C1, C7), medium rank bituminous coal (C2, C3, C6, C10), high coal rank bituminous coal (C4, C9) and anthracite (C5, C8). The inductively coupled plasma mass spectrometer (ICP-MS) was used to determine the iron, cobalt and nickel trace elements that affect biomethane generation in the coal seam (Table 5).

**Table 4:** Proximate analysis and Ultimate analysis

Coal no.	Location	Proximate analysis				Ultimate analysis				*R*_O_%
		*M*_ad_%	*A*_ad_%	*V*_ad_%	FC_ad_%	*C*_daf_%	*H*_daf_%	*N*_daf_%	(O + S)_daf_%	
C1	Yima	10.45	5.32	31.15	53.08	76.24	5.29	1.08	17.39	0.52
C2	Liyazhuang	0.77	17.77	23.34	58.12	83.23	5.58	1.25	9.94	0.80
C3	Shaqu	0.44	3.73	19.19	76.64	89.73	4.95	1.42	3.90	1.10
C4	Hebi	0.60	10.82	14.91	73.67	90.43	4.25	1.52	3.80	1.82
C5	Jingcheng	0.85	9.00	9.16	80.99	91.51	2.97	1.44	4.08	2.67
C6	Suzhou	1.43	20.50	25.85	52.22	87.28	5.42	1.40	5.90	0.84
C7	Neimeng	32.27	8.66	28.04	31.03	37.61	2.58	0.36	12.16	0.30
C8	Jiaozuo	0.63	8.30	12.22	78.85	91.67	2.67	1.16	4.39	3.15
C9	Shoushan	0.99	7.64	18.62	72.75	90.00	4.73	1.80	3.47	1.41
C10	Pingdingshan	0.73	8.01	24.20	67.06	86.50	5.50	1.38	6.62	0.95

**Table 5:** The content of Fe, Co and Ni in coal samples

Coal no.	Location	Fe/mg Kg^−1^	Co/μg Kg^−1^	Ni/μg Kg^−1^
C1	Yima	2235.33	1289.75	4477.59
C2	Liyazhuang	618.48	1417.73	3781.58
C3	Shaqu	421.90	925.38	4460.60
C4	Hebi	3308.47	1488.05	9126.36
C5	Jingcheng	3706.94	1947.78	5523.24
C6	Suzhou	4057.55	3892.16	12,117.65
C7	Neimeng	17,886.79	9459.12	32,816.75
C8	Jiaozuo	978.54	35,431.68	15,418.08
C9	Shoushan	3548.95	2410.61	10,014.18
C10	Pingdingshan	893.68	5825.13	15,301.62

### δ^13^C and δ^2^H for the methane

Table 6 shows that some of the areas studied have high CBM content and have been tested for carbon and hydrogen isotopes of methane Data show the presence of biogenic coalbed methane in some areas.

**Table 6:** The stable isotope characteristics of CBM in different regions

Coal no.	Location	δ^13^C_CH4_/‰	δD_CH4_/‰
C1	Yima		
C2	Liyazhuang	− 56.3 to − 61.7	
C3	Shaqu	− 42.28 to − 39.78	
C4	Hebi	− 57.44 to − 56.64	
C5	Jingcheng	− 40 to − 30	− 244 to − 145
C6	Suzhou (shallow)	− 63.74	
	Suzhou (deep)	− 47.27	
C7	Neimeng	− 73.2 to − 72.6	− 284 to − 280
C8	Jiaozuo	− 33.33 to − 35.79	
C9	Shoushan	− 36.13 to − 37.67	
C10	Pingdingshan		

All of these environmental factors, coal quality characteristics, and different origin of CBM will provide information for the future study of diversity of hydrogen-producing bacteria and methanogens in the coal seam in situ. The depth and reservoir temperature were measured in the sampling location and other information were obtained from the Geological data of local mines.

### Enrichment culture

Normally, microorganisms should be enriched until cell harvesting and DNA extraction; however, the methanogens and hydrogen-producing bacteria play a major role in this study, so selective medium was used for these microorganisms. Microorganisms must be cultured and acclimatized to increase gas production before biogas experiments. In this experiment, the following media were used to culture and enrich hydrogen-producing bacteria and methanogens from coal seam.

The hydrogen producing bacteria were cultured in (NH_4_Cl 1.0 g, MgCl_2_·6H_2_O 0.1 g, K_2_HPO_4_·3H_2_O 0.4 g, yeast extract 1 g, l-cysteine salt 0.5 g, NaHCO_3_ 2.0 g, glucose 10 g, tryptone 1.0 g, EDTA disodium salt 2.0 g) per liter.

Methanogens were cultured in (NH_4_Cl 1.0 g, MgCl_2_·6H_2_O 0.1 g, K_2_HPO_4_·3H_2_O 0.4 g, yeast extract 1 g, l-cysteine salt 0.5 g, Na_2_S 0.2 g, NaHCO_3_ 2.0 g, tryptone 0.1 g, sodium formate 2.0 g, sodium acetate 2.0 g) per liter.

In both cases, the prepared liquid culture medium was sterilized at 121 °C for 30 min, and then cooled and placed on sterilization counter. Fresh coal samples were carefully pulverized into 80 to 100 mesh and placed in liquid media under anaerobic conditions using an anaerobic workstation (DG250, UK). The coal samples were then cultured in the shaking incubator for 10–15 days at the same temperature as the sampling environment.

### DNA extraction and 16S rRNA gene amplification by PCR

Total community genomic DNA extraction was performed using a E.Z.N.A. Soil DNA Kit (Omega, USA), following the manufacturer’s instructions. We measured the concentration of the DNA using a Qubit 2.0 (life, USA) to ensure adequate high-quality genomic DNA had been extracted.

The primer pairs of 314 F (5′-cctacgggnggcwgcag-3′) and 805 R (5′-gactachvgggtatctaatcc-3′) have been demonstrated to have high coverages of all phyla in 16S rDNA microbiome analyses and were used to amplify the V3 and V4 regions of bacterial 16S rRNA genes [67]. The first primer pairs of 340F (5′-ccctayggggygcascag-3′) and 1000R (5′-ggccatgcacywcytctc-3′) and the second primer pairs of 349F (5′-gygcascagkcgmgaaw-3′) and 806R (5′-ggactacvsgggtatctaat-3′) were used to amplify the 16S rRNA genes of methanogenic archaea [68]. The reaction was set up as follows: microbial DNA (10 ng/μL) 2 μL; amplicon PCR forward primer (10 μM) 1 μL; amplicon PCR reverse primer (10 μM) 1 μL; 2× KAPA HiFi Hot Start Ready Mix 15 μL (total 30 μL). The plate was sealed and PCR performed in a thermal instrument (Applied Biosystems 9700, USA) using the following program: 1 cycle of denaturing at 95 °C for 3 min, first 5 cycles of denaturing at 95 °C for 30 s, annealing at 45 °C for 30 s, elongation at 72 °C for 30 s, then 20 cycles of denaturing at 95 °C for 30 s, annealing at 55 °C for 30 s, elongation at 72 °C for 30 s and a final extension at 72 °C for 5 min. The PCR products were checked using electrophoresis in 1% (w/v) agarose gels in TBE buffer (Tris, boric acid, EDTA) stained with ethidium bromide (EB) and visualized under UV light.

## 16S gene library construction and Illumina Miseq sequencing

Before sequencing, the DNA concentration of each PCR product was determined using a Qubit^®^ 2.0 Green double-stranded DNA assay and it was quality controlled using a bioanalyzer (Agilent 2100, USA). We used AMPure XP beads to purify the free primers and primer dimer species in the amplicon product. Samples were delivered to Sangon BioTech (Sangon Bio-Tech, Co., Ltd. Shanghai, China) for library construction using universal Illumina adaptor and index. The amplicons from each reaction mixture were pooled in equimolar ratios based on their concentration. Sequencing was performed using the Illumina MiSeq system (Illumina MiSeq, USA), according to the manufacturer’s instructions (Additional files 1, 2, 3).

### Data statistical analysis

Sequences were classified according to their barcodes, and FLASH was used to merge the paired sequences [69]. The maximum mismatch ratio of the overlap region was 0.1, and sequences with no matches were removed. The Prinseq was used to remove 200 bp short sequences [70], and the sliding window method was used to trimmed off bases belonging to reads tail, with quality value below 20 bp, and the length threshold value was 200 bp. Following this, amplification sequences obtained from non-target regions, barcodes and primers were deleted and sequencing error correction was performed using software of Mothur. The sample information after QC and Filter chimeras shown in Additional files 4.

The amplification sequences were clustered into operational taxonomic units (OTUs) at a similarity level of 97% using the clustering program Usearch [71], which were submitted to the RDP Classifier at a threshold value of 0.8 [72]. The alpha diversity, including the Shannon and Simpson index to evaluate the community diversity, the ACE and Chao1 index show the community richness, was calculated using the software package Mothur (version 1.30.1) [73]. The diversity of the community about this research can be seen in Additional file 5.

A Venn diagram was constructed to count intuitively the unique and shared number of OTUs between the samples. Principal component analysis (PCA) is a multivariate technique for simplifying data and hierarchical clustering analysis were performed to describe the Interrelationships of the microorganism communities in the coal samples. The PICRUSt was used to predict and analyze the composition of functional genes in samples according to the genetic function of sequencing microbial genomes has been established [74]. The abundance heat map of gene function prediction can be seen in Additional file 6. Redundancy analysis (RDA), an appropriate method for species with a linear model, was used to analyze the impact of the environment factors on the variance of the community structure. The R software (version 3.1.1) was used to conduct the RDA and PCA analysis.

## Additional files

**Additional file 1: Table S1.** The microorganism from different region were obtained through high-throughput sequencing.

**Additional file 2: Table S2.** The classification of methanogens from coal in different regions.

**Additional file 3: Table S3.** The classification of bacteria involved in producing hydrogen from coal samples in different areas.

**Additional file 4.** Sample information after QC.

**Additional file 5.** The diversity of community.

**Additional file 6.** The abundance heat map of gene function prediction.

## Abbreviations

CBM: coalbed methane
SRB: sulfate reducing bacteria
MECBM: microbial enhanced coalbed methane
VFA: volatile fatty acid
