# Supplementary material for: The diversity of hydrogen-producing bacteria and methanogens within an in situ coal seam
Source: Biotechnol Biofuels. 2018 Sep 8;11:245. doi: 10.1186/s13068-018-1237-2 (PMC6128992; doi:10.1186/s13068-018-1237-2)
Supplement: Supplementary file 1 — Additional file 1: Table S1. The microorganism from different region were obtained through high-throughput sequencing. [file 13068_2018_1237_MOESM1_ESM.docx]

Table S1. The microorganism from different region were obtained through high-throughput sequencing

| Affiliated taxon | Coal Samples | | | | | | | | | |
| --- | --- | --- | --- | --- | --- | --- | --- | --- | --- | --- |
|  | C1 | C2 | C3 | C4 | C5 | C6 | C7 | C8 | C9 | C10 |
| Euryarchaeota  Methanorix  Methanosarcina  Methanospirillum  Methanoculleus  Methanosphaerula  Methanoregula  Methanolinea  Methanobrevibacter  Methanobacterium  Methanothermobacter  Methanosphaera  Methanomassiliicoccus  Methanomethylovorans  Halakalicoccus  Methanolobus  Methanocella  Halovivax  Methanomicrobium  Methanosalsum  Natroncoccus  Methanofollis | 96.79  88.43  2.75  1.13  0.88  0.87  0.25  0.05  1.08  0.54  0.40  0.09  0.31 | 100  86.97  0.46  0.14  9.12  3.16  0.01 | 100  0.04  0.01  0.05  0.05  0.01  99.84  0.02 | 99.97  12.37  0.20  0.28  2.53  0.01  0.01  0.05  77.80  5.24  0.05  0.05  1.36  0.03 | 98.26  1.93  1.58  0.53  0.54  0.02  0.14  0.09  92.59  0.48  0.02  0.30  0.03 | 99.98  0.07  0.04  0.02  0.01  85.28  14.56 | 88.01  13.04  3.77  4.16  2.93  0.67  0.26  0.66  0.30  1.88  0.80  0.05  1.47  0.43  57.46 | 92.06  78..89  3.46  1.55  0.53  1.10  0.74  1.04  0.14  1.88  1.24  0.16  0.64  0.33  0.32 | 94.37  27.24  35.89  7.07  3.14  6.54  0.25  0.63  7.96  1.04  0.26  0.58  2.99  0.20  0.03  0.52 | 99.36  67.78  0.07  0.02  0.05  0.02  0.03  0.07  0.07  0.03  0.01  0.04  0.20  30.95 |
| Thaumarchaeota  Nitrososphaera  Nitrosopumilus | 0.20  0.20 |  |  | 0.01  0.01 | 0.05  0.05 |  | 0.55  0.55 | 0.62  0.53  0.09 | 0.94  0.89  0.04 | 0.09  0.09 |
| Woesearchaeota  Woesearchaeota  incertae Sedis AR16  AR15 | 0.04  0.04 |  |  |  | 0.01  0.01 |  | 0.29  0.29 |  | 0.05  0.05 | 0.01 |
| Pacearchaeota  Pacearchaeota incertae  Sedis AR13 | 0.06  0.06 |  |  |  |  |  |  |  |  | 0.01  0.01 |
| Alphaproteobacteria |  | 0.01 | 2.10 | 0.01 |  |  | 0.05 |  |  |  |
| Roseovarius |  | 0.01 |  |  |  |  |  |  |  |  |
| Brevundimonas |  |  | 0.95 |  |  |  |  |  |  |  |
| Caulobacter |  |  | 0.20 |  |  |  |  |  |  |  |
| Bosea |  |  | 0.21 |  |  |  |  |  |  |  |
| Bradyrhizobium |  |  |  | 0.01 |  |  |  |  |  |  |
| Sphingomonas |  |  |  |  |  |  |  |  |  |  |
| Magnetospinllum |  |  |  |  |  |  | 0.01 |  |  |  |
| Sulfitobacter |  |  |  |  |  |  | 0.01 |  |  |  |
| Betaproteobacteria |  |  | 1.38 |  |  | 0.02 |  |  | 0.04 | 0.06 |
| Aquabacterium |  |  | 0.32 |  |  |  |  |  |  |  |
| Ralstonia |  |  | 0.30 |  |  |  |  |  |  |  |
| Comamonas  Zoogloea  Hydrogenophaga  Thauera |  |  |  |  |  | 0.01 |  |  | 0.02  0.01 | 0.03  0.01  0.02 |
| Gammaproteobacteria | 46.18 | 0.04 | 17.13 | 57.49 |  | 93.20 | 0.54 | 65.91 | 57.91 | 0.24 |
| Pseudomonas | 0.02 |  | 5.84 | 0.01 |  | 0.02 | 0.10 | 0.11 | 0.01 | 0.05 |
| Pseudoalteromonas |  |  |  |  |  |  | 0.03 |  | 0.01 |  |
| Azomonas | 46.15 |  |  |  |  |  |  |  |  |  |
| Azotobacter |  |  |  |  |  |  |  |  |  |  |
| Serpens | 0.19 |  |  |  |  |  |  |  |  |  |
| Trabulslella |  |  |  | 0.76 |  |  | 0.01 |  |  |  |
| Pantoea |  | 0.02 |  |  |  |  |  |  |  |  |
| Acinetobacter |  |  | 1.63 |  |  |  | 0.13 | 22.49 |  | 0.04 |
| Psychrobacter |  | 0.01 | 0.38 |  |  |  |  |  |  |  |
| Enhydrobacter |  |  | 1.01 |  |  |  |  |  |  |  |
| Salmonella |  |  | 2.52 | 0.80 |  | 0.02 |  |  |  |  |
| Serratia |  |  | 0.24 | 0.30 |  |  |  |  |  |  |
| Stenotrophomonas |  |  | 2.88 |  |  |  |  |  |  |  |
| Klebsiella |  |  |  | 0.13 |  | 0.38 |  | 0.05 | 0.44 |  |
| Enterobacter |  |  | 0.81 | 1.97 |  | 1.18 |  | 9.77 | 1.86 | 0.01 |
| Aeromonas |  |  |  | 0.57 |  | 0.61 |  |  |  |  |
| Citrobacter |  |  | 1.13 | 50.91 |  | 67.79 | 0.28 | 13.06 | 55.52 | 0.13 |
| Escherichia |  |  |  |  |  | 10.47 |  |  |  |  |
| Kluyvera |  |  |  |  |  | 6.01 |  |  |  |  |
| Cronobacter |  |  |  |  |  | 5.52 |  |  |  |  |
| Salmonella |  |  |  |  |  |  |  |  |  |  |
| Erwinia |  |  |  |  |  |  |  | 11.90 |  |  |
| Pantoea |  |  |  |  |  | 0.03 |  | 4.88 |  |  |
| Leclercia |  |  |  | 0.84 |  | 0.27 |  | 0.37 | 0.14 |  |
| Epsilonproteobacteria |  |  |  |  |  |  |  | 0.03 |  |  |
| Arcobacter |  |  |  |  |  |  |  | 0.03 |  |  |
| Firmicutes | 53.69 | 72.47 | 77.79 | 42.49 | 99.90 | 6.76 | 99.31 | 33.88 | 42.04 | 99.31 |
| Pediococcus |  |  |  |  |  | 4.59 |  |  |  |  |
| Tissierella | 29.36 |  |  |  | 38.04 |  |  |  |  | 0.11 |
| Anaerosalibacter | 0.74 |  |  | 0.27 |  |  |  |  | 0.35 |  |
| Soehngenia | 0.01 |  |  |  |  |  |  |  |  |  |
| Clostridium sensu stricto  Clostridium XIVa  Clostridium XIVb  Clostridium IV  Clostridium III | 6.18  2.91  1.33  0.03 | 3.00  0.12  0.28 | 3.14 | 39.89  0.01 | 0.41  6.83 | 0.01  0.03  0.38 | 85.65  0.03  0.05 | 1.62  0.01 | 0.35  4.23  1.88  0.18 | 59.31 |
| Proteiniclasticum | 0.86 |  |  |  | 25.25 |  |  |  |  |  |
| Anaerobacter |  | 0.01 |  |  | 0.20 |  | 1.51 |  | 33.72 | 0.85 |
| Desulfosporosinus | 2.41 |  |  |  |  |  | 0.01 |  | 0.22 |  |
| Alkaliphilus | 2.23 |  |  |  | 0.01 |  |  |  |  |  |
| Oscillibacter | 0.97 |  |  |  |  |  |  |  |  |  |
| Lutispora  Acidaminobacter | 0.92 |  |  |  | 0.76 |  |  |  |  | 0.01 |
| Proteiniborus | 0.63 |  |  |  | 25.18 |  |  |  |  |  |
| Exiguobacterium | 1.57 | 0.05 |  |  |  | 0.39 | 0.42 | 0.34 | 0.04 | 0.14 |
| Bacillus | 0.38 | 0.62 | 10.33 |  | 0.07 | 0.01 | 11.46 | 31.25 | 0.09 | 3.17 |
| Paenibacillus | 0.02 |  | 20.34 | 0.01 | 0.06 |  |  |  | 0.21 |  |
| Enterococcus | 0.76 | 58.55 | 1.39 | 1.92 | 0.02 | 1.34 |  | 0.03 |  | 8.05 |
| Melissococcus |  | 0.01 |  |  |  |  |  |  |  |  |
| Acetonema | 0.05 |  |  |  |  |  |  |  |  |  |
| Sporolactobacillus |  | 8.26 |  | 0.14 |  |  |  |  |  |  |
| Lysinibacillus |  | 1.26 | 9.20 |  |  |  |  |  |  |  |
| Staphylococcus |  |  | 17.75 |  |  |  |  |  |  |  |
| Acetanaerobacterium | 1.60 |  |  |  |  |  |  |  |  |  |
| Brochothrix |  |  | 4.02 |  |  |  |  |  |  |  |
| Lactococcus |  |  | 9.96 |  | 0.01 |  | 0.01 | 0.01 |  |  |
| Natranaerovirga |  |  |  |  | 0.53 |  |  |  |  |  |
| Streptococcus |  |  | 0.54 |  |  |  |  |  |  |  |
| Leuconostoc |  |  | 0.36 |  |  |  |  |  |  |  |
| Carnobacterium |  |  | 0.14 |  |  |  |  |  |  |  |
| Intestinimonas |  |  |  |  | 0.76 |  |  |  |  |  |
| Ethanoligenens |  |  |  | 0.05 |  |  |  |  |  |  |
| Isobaculum |  | 0.23 |  | 0.17 |  |  |  |  |  | 0.01 |
| Sedimentibacter |  |  |  |  | 0.26 |  | 0.01 | 0.01 |  |  |
| Terrisporobacter |  |  |  |  | 19.50 |  |  |  |  |  |
| Geosporobacter |  |  |  |  | 2.66 |  |  |  | 0.01 | 0.09 |
| Desulfitobacterium |  |  |  |  |  |  | 0.11 |  |  |  |
| Allobacillus  Anoxybacillus |  |  |  |  |  |  |  | 0.50 |  | 27.37 |
| Bacteroidetes | 0.03 | 0.03 | 1.11 |  | 0.05 |  | 0.06 | 0.13 |  |  |
| Proteiniphilum |  |  |  |  | 0.02 |  | 0.01 |  |  |  |
| Petrimonas |  |  |  |  | 0.01 |  |  |  |  |  |
| Bacteroides |  |  |  |  | 0.01 |  |  | 0.04 |  |  |
| Sediminibacterium |  |  | 0.23 |  |  |  |  |  |  |  |
| Chishuiella |  |  | 0.20 |  |  |  |  |  |  |  |
| Chryseobacterium |  |  | 0.17 |  |  |  |  |  |  |  |
| Porphyromonas |  |  | 0.15 |  |  |  |  |  |  |  |
| Macellibacteroides |  | 0.01 | 0.16 |  |  |  |  | 0.04 |  |  |
| Alkallflexus |  |  |  |  |  |  |  | 0.01 |  |  |
| Actinobacteria | 0.08 | 27.42 |  |  | 0.01 |  | 0.01 | 0.02 |  | 0.38 |
| Euzebya |  |  |  |  |  |  |  | 0.01 |  |  |
| Arthrobacter |  |  |  |  |  |  | 0.01 |  |  |  |
| Bifidobacterium |  | 27.41 |  |  |  |  |  |  |  |  |
| Alloscardovia |  | 0.01 |  |  |  |  |  |  |  |  |
| Tessaracoccus | 0.04 |  |  |  |  |  |  |  |  | 0.13 |
| Propionicimonas  Propionibacterium | 0.03 |  |  |  |  |  |  |  |  | 0.26 |
| Chloroflexi |  |  |  |  |  |  | 0.02 |  |  |  |
| Levilinea |  |  |  |  |  |  | 0.01 |  |  |  |
| Acidobacteria |  | 0.01 |  |  |  |  |  |  |  |  |
