# Supplementary material for: The diversity of hydrogen-producing bacteria and methanogens within an in situ coal seam
Source: Biotechnol Biofuels. 2018 Sep 8;11:245. doi: 10.1186/s13068-018-1237-2 (PMC6128992; doi:10.1186/s13068-018-1237-2)
Supplement: Supplementary file 2 — Additional file 2: Table S2. The classification of methanogens from coal in different regions. [file 13068_2018_1237_MOESM2_ESM.docx]

Table S2 The classification of methanogens from coal in different regions.

| *Euryarchaeota* | *Methanomicrobia* | *Methanomicrobiales* | *Methanomicrobiaceae* | *Methanoculleus* | C1, C2, C3, C4, C5, C6, C7, C8, C9, C10 |
| --- | --- | --- | --- | --- | --- |
|  |  |  |  | *Methanofollis* | C10 |
|  |  |  | *Methanospirillaceae* | *Methanospirillum* | C1, C4, C5, C6, C7, C8, C9, C10 |
|  |  |  | *Methanoregulaceae* | *Methanosphaerula* | C1, C4、C5, C7, C9 |
|  |  |  |  | *Methanoregula* | C1, C2, C4, C7, C9, C10 |
|  |  |  |  | *Methanolinea* | C1, C2, C4, C5, C7, C8, C9 |
|  |  | *Methanosarcinales* | *Methanosarcinaceae* | *Methanosarcina* | C1, C3, C4, C5, C6, C7, C8, C9, C10 |
|  |  |  |  | *Methanolobus* | C2, C3, C4, C6, C7, C8, C9, C10 |
|  |  |  |  | *Methanomethylovorans* | C4, C6, C9 |
|  |  |  | *Methanotrichaceae* | *Methanothrix* | C1, C3, C4, C5, C6, C7, C8, C9, C10 |
|  |  | *Methanocellales* | *Methanocellaceae* | *Methanocella* | C8 |
|  | *Methanobacteria* | *Methanobacteriales* | *Methanobactericeae* | *Methanobacterium* | C1, C2, C4, C5, C6, C7, C9, C10 |
|  |  |  |  | *Methanosphaera* | C3, C4, C5, C6, C7, C9, C10 |
|  |  |  |  | *Methanobrevibacterium* | C4, C5, C6, C7, C9, C10 |
|  |  |  |  | *Methanothermobacter* | C5, C7, C9, C10 |
|  | *Thermoplasmata* | *Methanomassiliicoccales* | *Methanomassiliicoccaceaes* | *Methanomassiliicoccus* | C1, C4, C5, C6, C7, C8, C9, C10 |
|  | *Halobacteria* | *Halobacteriales* | *Halobacteriaceae* | *halobacterium* | C5, C7, C8, C9 |
