# Supplementary material for: The diversity of hydrogen-producing bacteria and methanogens within an in situ coal seam
Source: Biotechnol Biofuels. 2018 Sep 8;11:245. doi: 10.1186/s13068-018-1237-2 (PMC6128992; doi:10.1186/s13068-018-1237-2)
Supplement: Supplementary file 3 — Additional file 3: Table S3. The classification of bacteria involved in producing hydrogen from coal samples in different areas. [file 13068_2018_1237_MOESM3_ESM.docx]

Table S3. The classification of bacteria involved in producing hydrogen from coal samples in different areas.

| *Firmicutes* | *Clostridia* | *Clostridiales* | *Clostridiaceae*Ⅰ | *Clostridium sensu* | C1, C2, C3, C4, C5, C6, C7, C8, C9, C10 |
| --- | --- | --- | --- | --- | --- |
|  |  |  | *Clostridiaceae*Ⅱ | *Alkaliphilus* | C1, C5 |
|  |  |  | *Clostridiaceae incertae* | *Tissierella* | C1, C5, C10 |
|  |  |  |  | *Anaerobacter* | C1, C2, C5, C7, C9, C10 |
|  |  |  | *Lachnospiraceae* | *Clostridium* ⅪVa | C1, C2, C5, C6 |
|  |  |  | *Peptococcaceae* | *Desulfitobacterium* | C7 |
|  |  |  |  | *Desulfosporosinus* | C1, C7, C9 |
|  |  |  | *Rumincoccaceae* | *Acetanaerobacterium* | C1 |
|  |  |  |  | *Clostridium* Ⅳ | C1, C2, C4, C6, C7, C10 |
|  |  |  |  | *Clostridium* Ⅲ | C1, C8, C10 |
|  | *Bacilli* | *Bacillales* | *Bacillaceae* | *Bacillus* | C1, C2, C3, C5, C6, C7, C8, C9, C10 |
|  |  |  | *Paenibacillaceae* | *Paenibacillus* | C1, C3, C4, C5, C9 |
|  |  |  | *Bacillaceae incertae* | *Exiguobacterium* | C1, C2, C6, C7, C8, C9, C10 |
|  |  | *Lactobacillales* | *Lactobacillaceae* | *Lactobacillus* | C7 |
|  |  |  | *Enterococcaceae* | *Enterococcus* | C1, C2, C3, C4, C5, C6, C8, C10 |
| *Proteobacteria* | *Γ-Proteobacteria* | *Pseudomonadales* | *Pseudomonadaceae* | *Azomonas* | C1 |
|  |  |  |  | *Pseudomonas* | C1, C3, C4, C6, C7, C8, C9, C10 |
|  |  |  | *Enterobacteriaceae* | *Enterobacter* | C3, C4, C6, C8, C9, C10 |
|  |  |  |  | *Salmonella* | C3, C4, C6 |
|  |  |  |  | *Citrobacter* | C3, C4, C6, C7, C8, C9, C10 |
|  |  |  |  | *Klebsiella* | C4, C6, C8, C9 |
|  |  |  | *Moraxellaceae* | *Acinetobacter* | C3、C7、C8、C10 |
|  | *Alphaproteobacteria* | *Rhodobacterales* | *Rhodobacteraceae* | *Roseobacter* | C10 |
|  |  | *Caulobacterales* | *Caulobacteraceae* | *Sulfitobacter* | C7、C8 |
|  |  |  |  | *Brevundimonas* | C3 |
|  |  |  |  | *Caulobacter* | C3 |
|  |  | *Rhizobiales* | *Bradyrhizobiaceae* | *Bosea* | C3 |
|  |  |  |  | *Rhizobium* | C7 |
| *Bacteroidetes* | *Bacteroidetes* | *Bacteroidales* | *Porphyromonadaceae* | *Macellibacteroides* | C2, C3, C8 |
|  |  |  | *Bacteroidaceae* | *Bacteroides* | C5, C8 |
|  |  |  | *Marinilabiliaceae* | *Alkaliflexus* | C8 |
